# Dark Triad, Depression, Anhedonia and Alexithymia: The Role of Sex Differences

**DOI:** 10.3390/bs15101369

**Published:** 2025-10-07

**Authors:** Daniel French, Gwenolé Loas, Matthieu Hein

**Affiliations:** 1 Service de Psychiatrie et Laboratoire du Sommeil, Hôpital Universitaire de Bruxelles, Université Libre de Bruxelles, 1070 Bruxelles, Belgium; daniel.french@hubruxelles.be; 2 Faculté de Médecine, Université Libre de Bruxelles, 1070 Bruxelles, Belgium; gwenole.loas@ulb.be; 3 Service de Psychiatrie et Laboratoire du Sommeil, CHU Brugmann, Université Libre de Bruxelles, 1020 Bruxelles, Belgium; 4 Laboratoire de Psychologie Médicale et Addictologie (ULB312), Université Libre de Bruxelles, 1020 Bruxelles, Belgium

**Keywords:** Dark Triad, alexithymia, anhedonia, depression, sex differences, emotional regulation

## Abstract

The Dark Triad (Machiavellianism, narcissism, and psychopathy) has been traditionally associated with externalizing behaviors and a lack of empathy. However, emerging evidence suggests that these traits also relate to internal emotional vulnerabilities (such as depression, alexithymia, and anhedonia). This study aimed to examine the association between Dark Triad traits and emotional variabilities (alexithymia and anhedonia) in the general population, with a particular focus on sex differences. A total of 492 French-speaking adults completed a battery of validated self-report measures online, including the SD3fr, BDI-II, TAS-20, and PAS. Comparative and multivariate analyses were conducted separately for males and females. High SD3 scores were associated with greater depression, alexithymia (especially difficulty in identifying feelings), and anhedonia in the overall sample. These associations remained significant after adjusting for depression severity. Sex differences emerged: in females, high Dark Triad traits were strongly linked to emotional dysfunction, while no such associations were observed in males. These findings support the presence of sex-specific emotional correlates of the Dark Triad, particularly in females, where Machiavellianism and psychopathy were linked to emotional distress. Clinical implications are discussed in light of hormonal, sociocultural, and emotional regulation differences.

## 1. Introduction

The Dark Triad is a group of three personality traits: Machiavellianism (characterized by a marked tendency to manipulate and exploit others for one’s own benefit without regard for morality), narcissism (characterized by an exclusive love of self, an overestimation of one’s own importance, a desire for power, a lack of empathy, and a tendency toward selfishness], and psychopathy (characterized by a “cold” affective core [lack/absence of empathy or remorse) and antisocial behaviors) (Kowalski et al., 2021). However, narcissism measured in most Dark Triad trait assessment tools is “grandiose” narcissism, characterized by a sense of superiority and self-enhancement that does not consider the potential psychological vulnerabilities linked to this personality trait (Maples et al., 2014).

One of the main features of individuals with Dark Triad traits is a lack of self-control induced by their higher impulsivity that may favor the occurrence of aggressive and criminal behaviors (Jones & Paulhus, 2014; Pechorro et al., 2022). It has been demonstrated that individuals with Dark Triad traits exhibit a lack of remorse with significant antisocial and criminal tendencies (Brugués & Caparrós, 2021; Navas et al., 2021; Hurezan et al., 2024). While most available studies have focused mainly on the negative impacts of Dark Triad traits on society (aggressiveness and a tendency to commit acts that violate norms) (Hampejs et al., 2025; Hurezan et al., 2024), some arguments have gradually emerged in the literature in favor of the existence of psychological suffering associated with Dark Triad traits (depressive symptoms, anxious manifestations, abnormal stress reactivity, sleep disturbances, and lack of well-being) (Coleman et al., 2019; J. Li et al., 2024; Sabouri et al., 2016; Gómez-Leal et al., 2023). Nevertheless, alongside these interesting preliminary data, some evidence indicates that “grandiose” narcissism, as typically measured in Dark Triad trait assessment tools, may have a protective effect against psychological distress, unlike the other two components of the Dark Triad (Papageorgiou et al., 2023). Among the mechanisms potentially involved in the occurrence of this psychological suffering associated with some components of the Dark Triad, the existence of maladaptive emotional regulation strategies appears to play a central role in individuals with Dark Triad traits (Mojsa-Kaja et al., 2021; Shen, 2022). However, despite their potential negative impacts on psychological functioning, complaints of alexithymia (difficulty identifying, differentiating, and expressing emotions) and anhedonia (inability to feel positive emotions during life situations previously considered pleasant) that may be induced by these maladaptive emotional regulation strategies have been poorly studied in individuals with Dark Triad traits (Cairncross et al., 2013; Garofalo et al., 2019; Grigoras & Wille, 2017; Loeffler et al., 2020). Given these different elements, it could be valuable to investigate the relationship between the presence of Dark Triad traits, paying particular attention to their specific “grandiose” narcissism dimension and the occurrence of some emotional dysfunctions (alexithymia and anhedonia) to allow for a better understanding of the potential impact of these personality traits on emotional functioning in the general population. It is theoretically plausible to expect that only Machiavellianism and psychopathy will correlate with emotional dysfunctions such as alexithymia and anhedonia, whereas grandiose narcissism will not.

Recent theoretical and empirical advances have further clarified the nature and implications of the Dark Triad traits. While originally conceptualized as a cluster of socially specific traits, current research emphasizes both their shared core and distinct psychological profiles (Book et al., 2015). In particular, Book et al. (2015) argued for the centrality of callousness and manipulation as a common core, while still recognizing differential expressions across Machiavellianism, narcissism, and psychopathy. Moreover, cross-cultural research by Jonason et al. (2020) has demonstrated that the prevalence and manifestation of Dark Triad traits are not culturally uniform but vary significantly depending on societal-level factors, such as inequality and cultural individualism, reinforcing the need for culturally sensitive frameworks. Developmental perspectives also bring new insights: Pu et al. (2025) suggested that early experiences of social ostracism and loneliness may contribute to the development of these traits in adolescents, highlighting the importance of contextual and interpersonal variables in the emergence of Dark Triad traits. Finally, Schreiber et al. (2020) conducted a meta-analytic synthesis to locate the Dark Triad within broader personality models, concluding that these traits are best understood as maladaptive variants of established dimensions within the Five-Factor Model. Together, these studies underline the theoretical richness and complexity of the Dark Triad and justify the need to contextualize specific hypotheses, such as their links to emotional dysfunctions or sex differences, in this study.

Several tools have been developed to assess Dark Triad traits, among which the Short Dark Triad (SD3) and the Dirty Dozen (DD) are the most frequently used (Maples et al., 2014). The SD3 is often preferred due to its broader and more nuanced coverage of each trait. Compared with the DD, the SD3 includes a wider range of items per dimension (9 items vs. 4 per trait), improving the specificity and discriminant validity of each subscale. It offers a more granular assessment of grandiose narcissism, which is often confounded with vulnerable narcissism in the DD. Studies have also shown that the SD3 demonstrates slightly stronger psychometric properties, including better internal consistency and test–retest reliability, especially in non-clinical adult populations (Maples et al., 2014; Dragostinov & Mõttus, 2023). These advantages justify the use of the SD3 in this study, especially in a French-speaking context where the scale has been translated and validated.

Sex is a central variable to consider when studying Dark Triad traits (Muris et al., 2017). It has been shown that males scored higher than females in all three dimensions of the Dark Triad (Machiavellianism, narcissism, and psychopathy) (Luo et al., 2023). Moreover, this difference between males and females is also present when focusing on “grandiose” narcissism, which is typically measured when assessing Dark Triad traits (Grijalva et al., 2015). Moreover, these differences between males and females for Dark Triad traits are consistent regardless of individuals’ culture (Schmitt et al., 2017). Furthermore, in case of stressful life events, there are arguments for different emotional regulation strategies between males and females (Duarte et al., 2015; Luque et al., 2020; Nolen-Hoeksema, 2012; Ricarte Trives et al., 2016). Furthermore, it was found that females had a greater variety of emotional regulation strategies than males (Duarte et al., 2015; Luque et al., 2020; Nolen-Hoeksema, 2012; Ricarte Trives et al., 2016). However, this difference between males and females is not limited only to adaptive emotional regulation strategies, but also exists in the case of maladaptive emotional regulation strategies (Duarte et al., 2015; Nolen-Hoeksema, 2012; Ricarte Trives et al., 2016). Compared with males, females may resort to ruminations more frequently in the case of maladaptive emotional regulation strategies (Duarte et al., 2015; Nolen-Hoeksema, 2012; Ricarte Trives et al., 2016), which favors the development of a “vulnerable” rather than a “grandiose” narcissism (Green et al., 2022). However, these sex-related differences in the occurrence of potential maladaptive emotional regulation strategies may be associated with a different expression of some emotional dysfunctions between males and females (Welkie et al., 2021).

It has been shown, in both the general population and some psychiatric subpopulations, that complaints of alexithymia and anhedonia tend to be expressed differently between males and females (Adewuya et al., 2018; Amerio et al., 2023; Mendia et al., 2024; Parker et al., 2014). These sex-specific emotional-processing patterns may contribute to different manifestations of emotional dysfunctions in males and females with similar Dark Triad traits. Several hypotheses were formulated a priori to guide our investigation of potential sex-specific patterns. From a biological perspective, hormones such as testosterone and estradiol have been associated with emotional regulation and antisocial traits, and may partially account for the differential manifestations of Dark Triad traits across sex (Albert et al., 2021). Sociocultural frameworks also suggest that traditional male sex roles—emphasizing power, control, and emotional restraint—may foster higher levels of Machiavellianism and psychopathy, while simultaneously discouraging emotional expression and awareness (Krampen et al., 1990; Gómez-Leal et al., 2019). Furthermore, theories of emotional regulation propose that females with high psychopathic traits may more frequently exhibit internalizing symptoms such as depression or alexithymia, potentially due to earlier traumatic experiences or conflict with socially prescribed sex norms (Fournier & Verona, 2022; Garofalo et al., 2021; Velotti et al., 2016). In this context, it therefore seems necessary to study sex differences for Dark Triad traits, paying particular attention to their specific “grandiose” narcissism dimension and their impacts on the expression of some emotional dysfunctions (alexithymia and anhedonia) associated with these personality traits, in order to identify the potential effects of sex on emotional functioning among individuals with Dark Triad traits. Given the data available in the literature, the “grandiose” narcissism associated with the Dark Triad would be characterized by a protective effect against psychological suffering.

This study, therefore, aimed to explore whether sex modulates the association between Dark Triad traits and emotional difficulties, shedding light on possible sex-specific pathways linking personality traits and affective processes. Thus, our study examined two primary hypotheses: 1/In the studied population, Machiavellianism and psychopathy—unlike grandiose narcissism—will be associated with higher levels of alexithymia and anhedonia; and 2/these associations will differ by sex, reflecting underlying differences in emotional regulation strategies and sex socialization. This approach aimed to open new perspectives for the development of more personalized care for individuals with Dark Triad traits, thanks to better identification of emotional dysfunctions present depending on sex.

## 2. Materials and Methods

### 2.1. Sample Recruitment

The 492 individuals included in this study were recruited prospectively using an online questionnaire on the LimeSurvey platform. This questionnaire was distributed between 11 July 2017 and 16 August 2018 via the social network Facebook, an email chain among students/staff members of the Université Libre de Bruxelles, and word of mouth. The sample was thus drawn from a community population, not a clinical one. For this study, the inclusion criteria were as follows: sufficient understanding of French and the ability to give informed consent. Meanwhile, the exclusion criteria were as follows: age < 18 years and missing data in the online questionnaire. In this study, given that the objective was to obtain a community sample, strict exclusion criteria were not applied in order to be as representative as possible.

### 2.2. Online Survey Description

The online survey consisted of a collection of demographic data and a series of self-administered questionnaires:

The subclinical presence of Dark Triad traits was studied using the Short Dark Triad (SD3). This scale consists of 27 items measuring the three traits of the Dark Triad: narcissism (9-item subscale), Machiavellianism (9-item subscale), and psychopathy (9-item subscale). Each item is scored from 1 (strongly disagree) to 5 (strongly agree). The presence of a high total score indicates a high level of subclinical Dark Triad traits. The same principle applies to the interpretation of the score of the three subscales of the Short Dark Triad (narcissism, Machiavellianism, and psychopathy). Given the absence of validated cut-offs for the SD3, the median was used to define the presence of high SD3 scores for males (score ≥ 78) and females (score ≥ 70) included in this study. This choice to use a different cut-off for males and females was made following the existence of data in favor of higher scores in males than in females for the SD3. The French translation of the SD3 used in this study was previously validated psychometrically (French et al., 2024). In that validation study, a confirmatory factor analysis supported a model with three correlated latent factors corresponding to Machiavellianism, narcissism, and psychopathy. This factorial structure was consistent with the original theoretical model proposed by Paulhus and Williams (2002), which conceptualizes the Dark Triad as comprising three distinct but interrelated constructs. Furthermore, for this study, the psychometric properties of the French version of the SD3 used were checked based on the specific data from our sample via internal consistency analyses based on Cronbach’s alpha and confirmatory factor analyses (Supplementary Data S1). These different analyses highlighted the adequate psychometric properties of the French version of the SD3 used in this study, with similar results to those of the previous validation study in French (French et al., 2024). As such, the French version of the SD3 used in this study aligns with the established theoretical structure of the Dark Triad (French et al., 2024).

Alexithymia was investigated using the Toronto Alexithymia Scale (TAS-20). This scale consists of 20 items divided into three subscales: the difficulty describing feelings (DDF) subscale (5 items), the difficulty identifying feelings (DIF) subscale (7 items), and the externally oriented thinking (EOT) subscale (8 items). Each item of this scale may be scored from “strongly agree” to “strongly disagree” on a five-point Likert scale. Three different levels of alexithymia are considered: non-alexithymic (scores of less than 51), moderately alexithymic (scores between 51 and 60), and severely alexithymic (scores over 60) (Loas et al., 1997).

Trait anhedonia was investigated using the Consumptive and Anticipatory Anhedonia Questionnaire derived from the revised Physical Anhedonia Scale of Chapman. This 26-item questionnaire consists of two subscales: a consumptive trait anhedonia subscale (16 items) and an anticipatory trait anhedonia subscale (10 items). Each item may be scored 0 or 1, which provides a total score as well as a score for each of the two subscales. Higher scores indicate higher levels of trait anhedonia (Loas et al., 2009).

Recent changes in anhedonia were studied using the anhedonia subscale of the Beck Depression Inventory (BDI-II). This subscale of recent changes in anhedonia, validated by Joiner et al. (2003), contains items 4, 12, and 21 from the Beck Depression Inventory (BDI-II) (Joiner et al., 2003).

The presence of depressive symptoms was investigated using the Beck Depression Inventory (BDI-II). This scale consists of 21 items that may be scored from 1 to 3. The score may vary from 0 to 63. A score of 0–9 indicates no depression, 10–18 indicates mild depression, 19–29 indicates moderate depression, and 30–63 indicates severe depression (Beck et al., 1996).

These self-report questionnaires were available in the online survey in the order described in this study. This order was defined based on the importance of the subjects’ responses to these questionnaires to achieve this study’s objective.

### 2.3. Statistical Analyses

The Stata 14 software was used to perform the statistical analyses in this study. Histograms, boxplots, and quantile–quantile plots were used to check the distribution of continuous data. Since parametric tests were not usable for some of the continuous data in this study after checking their distribution and equality of variances (Conenna et al., 2025), non-parametric tests based on the median (Wilcoxon tests) were performed to highlight significant differences between groups with low SD3 scores and groups with high SD3 scores. Subsequently, multivariate quantile regression models (based on the median) were used to adjust our results for the presence of depressive symptoms (Beck Depression Inventory scores). In these models, emotional variables were considered as the dependent variables, whereas depressive symptoms (Beck Depression Inventory scores) were used as predictors to adjust regression coefficients between groups with low SD3 scores and groups with high SD3 scores. This adjustment for the presence of depressive symptoms (Beck Depression Inventory scores) was decided following the presence of significant differences for this variable between groups with low SD3 scores and groups with high SD3 scores. The presence of depressive symptoms is one of the main factors associated with the occurrence of complaints of alexithymia and anhedonia in the general population (Honkalampi et al., 2000; Damerdji et al., 2022; S. Li et al., 2015; Serretti, 2023), which justifies considering this potential major confounding factor for adjusting the results of this study. These different analyses were carried out separately for the whole sample, including females and males.

For categorical data, comparison analyses between groups with low SD3 scores and groups with high SD3 scores were performed using Chi^2^ tests. Spearman correlations were used for correlation analyses between Dark Triad traits and emotional variables. Results were considered significant when the *p*-value was <0.05.

Participants with missing data on any of the self-report scales were excluded from the different analyses, allowing for the performance of different analyses on the same sample without variation in the number of subjects depending on the variable studied.

## 3. Results

### 3.1. Descriptive Statistics

The sample consisted of 492 participants, including 323 females and 169 males. The median age was 29 years (IQR: 26–33). Regarding professional activities, 17.3% were students or young workers, 76.8% were adult workers, and 5.9% were middle-aged to older workers. For educational level, 64.8% of the subjects had a higher secondary education diploma or a higher education diploma, unlike the other 35.2% of subjects from the sample. These descriptive characteristics are presented in Table 2 for the whole sample, Table 3 for females and Table 4 for males. No statistically significant differences were observed between individuals with high versus low SD3 scores regarding age, professional activity, or educational level for the whole sample, including females and males. A detailed description of the different variables studied according to age ranges is available in the Supplementary Data S2.

### 3.2. SD3 Variables—Females and Males (Table 1)

Compared with females, males had higher scores on the SD3 total [78 (IQR: 71–87) for males vs. 70 (IQR: 62–78) for females; *p* < 0.001], the SD3 Machiavellianism subscale [30 (IQR: 26–35) for males vs. 27 (IQR: 23–31) for females; *p* < 0.001], the SD3 narcissism subscale [26 (IQR: 23–30) for males vs. 24 (IQR: 21–27) for females; *p* < 0.001], and the SD3 psychopathy subscale [22 (IQR: 19–26) for males vs. 19 (IQR: 16–22) for females; *p* < 0.001].

**Table 1:** Comparative analyses for SD3 variables between females and males (*n* = 492).

Variables	Whole Sample (*n* = 492)	Females (*n* = 323)	Males (*n* = 169)	*p*-Value
SD3—Machiavellianism	28 (24–32)	27 (23–31)	30 (26–35)	<0.001
SD3—Narcissism	25 (21–28)	24 (21–27)	26 (23–30)	<0.001
SD3—Psychopathy	20 (17–23)	19 (16–22)	22 (19–26)	<0.001
SD3—Total	72 (65–82)	70 (62–78)	78 (71–87)	<0.001

SD3 = Short Dark Triad.

### 3.3. Comparative Analyses for Whole Sample

#### 3.3.1. Confounding Variables (Table 2)

Individuals (males and females) with high SD3 scores had higher Beck Depression Inventory scores [BDI: 12 (IQR: 6–18); *p* = 0.002] than those with low SD3 scores [BDI: 9 (IQR: 4–17); *p* = 0.002]. There were no significant differences for age and sex.

**Table 2:** Comparative analyses for whole sample (*n* = 492).

Variables	Whole Sample (*n* = 492)	Females + Males with Low SD3 Scores (*n* = 235)	Females + Males with High SD3 Scores (*n* = 257)	*p*-Value
*SD3 Variables*				
SD3—Machiavellianism	28 (24–32)	24 (22–27)	31 (28–35)	<0.001
SD3—Narcissism	25 (21–28)	22 (19–24)	27 (24–31)	<0.001
SD3—Psychopathy	20 (17–23)	17 (15–20)	22 (20–26)	<0.001
SD3—Total	72 (65–82)	65 (58–68)	81 (75–87)	<0.001
*Emotional variables*				
TAS—DIF	17 (13–22)	16 (12–21)	18 (14–23)	<0.001
TAS—DDF	14 (10–18)	13 (10–17)	14 (11–18)	0.050
TAS—EOT	16 (14–19)	16 (13–19)	16 (14–19)	0.288
TAS—Total	48 (39–56)	45 (37–54)	50 (42–58)	<0.001
Trait anhedonia—Anticipatory	5 (5–6)	5 (5–6)	5 (5–6)	0.744
Trait anhedonia—Consummatory	6 (5–7)	6 (5–8)	6 (5–7)	0.854
Trait anhedonia—Total	11 (10–13)	11 (10–13)	11 (10–13)	0.738
Recent change in anhedonia	1 (0–2)	1 (0–2)	1 (0–2)	0.007
*Confounding variables*				
Age (years)	29 (26–33)	29 (26–34)	29 (26–32)	0.610
Sex (M)	34.3%	33.6%	35.0%	0.744
BDI—Total	10 (5–18)	9 (4–17)	12 (6–18)	0.002
Professional activities				0.713
Student or young workers	17.3%	17.4%	17.1%	
Adult working population	69.5%	68.1%	70.8%	
Middle-aged to older working population	13.2%	14.5%	12.1%	
Education level				0.905
Other levels of education	35.2%	34.9%	35.4%	
Upper secondary or higher education	64.8%	65.1%	64.6%	

SD3 = Short Dark Triad, TAS = Toronto Alexithymia Scale, DIF = difficulty identifying feelings, DDF = difficulty describing feelings, EOT = externally oriented thinking, BDI = Beck Depression Inventory.

#### 3.3.2. Emotional Variables (Table 2)

Compared with those with low SD3 scores, individuals (males and females) with high SD3 scores had higher scores on the TAS-20 total [50 (IQR: 42–58) vs. 45 (IQR: 37–54); *p* < 0.001], the TAS-20 DIF subscale [18 (IQR: 14–23) vs. 16 (IQR: 12–21); *p* < 0.001], and the anhedonia subscale of the Beck Depression Inventory [1 (IQR: 0–2) vs. 1 (IQR: 0–2); *p* = 0.007). These differences remained significant after adjustment for Beck Depression Inventory scores (Supplementary Data S3—Table S1). There were no significant differences for other emotional variables.

### 3.4. Comparative Analyses for Females

#### 3.4.1. Confounding Variables (Table 3)

Females with high SD3 scores had higher Beck Depression Inventory scores [BDI: 13 (IQR: 7–19); *p* = 0.001] than those with low SD3 scores [BDI: 9 (IQR: 3–17); *p* = 0.001]. There were no significant differences for age.

**Table 3:** Comparative analyses for females (*n* = 323).

Variables	Whole Sample (*n* = 323)	Individuals with Low SD3 Scores (*n* = 156)	Individuals with High SD3 Scores (*n* = 167)	*p*-Value
*SD3 Variables*				
SD3—Machiavellianism	27 (23–31)	23 (20–26)	31 (28–33)	<0.001
SD3—Narcissism	24 (21–27)	22 (19–24)	26 (23–29)	<0.001
SD3—Psychopathy	19 (16–22)	16 (14–18)	21 (19–23)	<0.001
SD3—Total	70 (62–78)	62 (56–66)	77 (72–83)	<0.001
*Emotional variables*				
TAS—DIF	17 (13–22)	16 (12–20)	19 (14–24)	<0.001
TAS—DDF	13 (10–17)	12 (9–16)	13 (10–18)	0.051
TAS—EOT	16 (13–19)	16 (13–19)	16 (14–19)	0.232
TAS—Total	47 (38–55)	44 (36–53)	50 (41–57)	<0.001
Trait anhedonia—Anticipatory	5 (5–6)	5 (5–6)	5 (5–6)	0.992
Trait anhedonia—Consummatory	6 (5–7)	6 (5–7)	6 (5–7)	0.695
Trait anhedonia—Total	11 (10–13)	11 (10–13)	11 (10–13)	0.873
Recent change in anhedonia	1 (0–2)	1 (0–2)	1 (0–3)	0.003
*Confounding variables*				
Age (years)	29 (26–33)	29 (26–34)	29 (26–32)	0.957
BDI—Total	11 (5–18)	9 (3–17)	13 (7–19)	0.001
Professional activities				0.723
Student or young workers	16.4%	18.0%	15.0%	
Adult working population	71.2%	69.2%	73.0%	
Middle-aged to older working population	12.4%	12.8%	12.0%	
Education level				0.754
Other levels of education	33.1%	34.0%	32.3%	
Upper secondary or higher education	66.9%	66.0%	67.7%	

SD3 = Short Dark Triad, TAS = Toronto Alexithymia Scale, DIF = difficulty identifying feelings, DDF = difficulty describing feelings, EOT = externally oriented thinking, BDI = Beck Depression Inventory.

#### 3.4.2. Emotional Variables (Table 3)

Compared with those with low SD3 scores, females with high SD3 scores had higher scores on the TAS-20 total [50 (IQR: 41–57) vs. 44 (IQR: 36–53); *p* < 0.001], the TAS-20 DIF subscale [19 (IQR: 14–24) vs. 16 (IQR: 12–20); *p* < 0.001], and the anhedonia subscale of the Beck Depression Inventory [1 (IQR: 0–2) vs. 1 (IQR: 0–3); *p* = 0.003]. These differences remained significant after adjustment for Beck Depression Inventory scores (Supplementary Data S3—Table S2). There were no significant differences for other emotional variables.

### 3.5. Comparative Analyses for Males

#### 3.5.1. Confounding Variables (Table 4)

There were no significant differences for age and Beck Depression Inventory scores between males with high SD3 scores and those with low SD3 scores.

**Table 4:** Comparative analyses for males (*n* = 169).

Variables	Whole Sample (*n* = 169)	Individuals with Low SD3 Scores (*n* = 79)	Individuals with High SD3 Scores (*n* = 90)	*p*-Value
*SD3 Variables*				
SD3—Machiavellianism	30 (26–35)	27 (24–30)	35 (30–37)	<0.001
SD3—Narcissism	26 (23–30)	23 (20–25)	30 (26–33)	<0.001
SD3—Psychopathy	22 (19–26)	19 (16–22)	25 (22–28)	<0.001
SD3—Total	78 (71–87)	71 (65–74)	87 (82–94)	<0.001
*Emotional variables*				
TAS—DIF	17 (14–21)	16 (12–21)	18 (14–22)	0.121
TAS—DDF	15 (12–19)	15 (11–18)	16 (12–19)	0.441
TAS—EOT	16 (14–19)	16 (14–19)	16 (14–19)	0.888
TAS—Total	49 (41–57)	47 (40–55)	50 (42–58)	0.234
Trait anhedonia—Anticipatory	5 (4–6)	5 (4–6)	5 (4–6)	0.637
Trait anhedonia—Consummatory	7 (5–8)	7 (5–8)	6 (5–8)	0.430
Trait anhedonia—Total	12 (10–13)	11 (10–13)	12 (10–13)	0.490
Recent change in anhedonia	1 (0–2)	1 (0–2)	1 (0–2)	0.663
*Confounding variables*				
Age (years)	29 (26–32)	30 (26–34)	29 (25–32)	0.414
BDI—Total	10 (5–16)	10 (5–16)	10 (5–16)	0.778
Professional activities				0.510
Student or young workers	18.9%	16.5%	21.1%	
Adult working population	66.3%	65.8%	66.7%	
Middle-aged to older working population	14.8%	17.7%	12.2%	
Education level				0.558
Other levels of education	39.0%	36.7%	41.1%	
Upper secondary or higher education	61.0%	63.3%	58.9%	

SD3 = Short Dark Triad, TAS = Toronto Alexithymia Scale, DIF = difficulty identifying feelings, DDF = difficulty describing feelings, EOT = externally oriented thinking, BDI = Beck Depression Inventory.

#### 3.5.2. Emotional Variables (Table 4)

There were no significant differences for emotional variables between males with high SD3 scores and those with low SD3 scores (Supplementary Data S3—Table S3 for adjusted comparative analyses).

### 3.6. Correlation Analyses

#### 3.6.1. For Females (Table 5)

Total SD3 scores were positively correlated with TAS-20 DIF subscale scores (rs = 0.307; *p* < 0.05), TAS-20 total scores (rs = 0.284; *p* < 0.05), and anhedonia subscale of Beck Depression Inventory scores (rs = 0.143, *p* < 0.05). There were no significant correlations between total SD3 scores and other emotional variables.

**Table 5:** Correlation analyses between SD3 and emotional variables for females and males (*n* = 492).

Variables	SD3—Total	SD3—Machiavellianism	SD3—Narcissism	SD3—Psychopathy
*Whole sample*				
TAS—DIF	0.242 ^a^	0.206 ^a^	0.104	0.291 ^a^
TAS—DDF	0.187 ^a^	0.241 ^a^	−0.031	0.238 ^a^
TAS—EOT	0.098	0.133	−0.049	0.142
TAS—Total	0.247 ^a^	0.263 ^a^	0.031	0.301 ^a^
Trait anhedonia—Anticipatory	−0.088	−0.077	−0.097	−0.041
Trait anhedonia—Consummatory	0.026	0.083	−0.019	−0.025
Trait anhedonia—Total	−0.023	0.030	−0.076	−0.026
Recent change in anhedonia	0.143 ^a^	0.177 ^a^	−0.002	0.154 ^a^
*Females*				
TAS—DIF	0.307 ^a^	0.268 ^a^	0.099	0.360 ^a^
TAS—DDF	0.173	0.243 ^a^	−0.117	0.268 ^a^
TAS—EOT	0.133	0.190 ^a^	−0.059	0.181 ^a^
TAS—Total	0.284 ^a^	0.308 ^a^	−0.001	0.356 ^a^
Trait anhedonia—Anticipatory	−0.028	−0.016	−0.101	0.017
Trait anhedonia—Consummatory	0.010	0.067	−0.013	−0.061
Trait anhedonia—Total	−0.020	0.029	−0.078	−0.038
Recent change in anhedonia	0.214 ^a^	0.204 ^a^	0.018	0.257 ^a^
*Males*				
TAS—DIF	0.172	0.122	0.116	0.218
TAS—DDF	0.121	0.167	0.023	0.105
TAS—EOT	0.006	0.016	−0.076	0.078
TAS—Total	0.146	0.155	0.030	0.190
Trait anhedonia—Anticipatory	−0.023	−0.033	0.002	0.012
Trait anhedonia—Consummatory	0.002	0.092	−0.075	−0.012
Trait anhedonia—Total	0.004	0.074	−0.062	0.023
Recent change in anhedonia	0.062	0.166	−0.031	0.015

^a^ *p* < 0.05. TAS = Toronto Alexithymia Scale, DIF = difficulty identifying feelings, DDF = difficulty describing feelings, EOT = externally oriented thinking.

SD3 Machiavellianism/psychopathy subscale scores were positively correlated with TAS-20 DIF subscale scores (rs = 0.268; *p* < 0.05), TAS-20 DDF subscale scores (rs = 0.243; *p* < 0.05), TAS-20 EOT subscale scores (rs = 0.190; *p* < 0.05), TAS-20 total scores (rs = 0.308; *p* < 0.05), and anhedonia subscale of Beck Depression Inventory scores (rs = 0.177; *p* < 0.05). There were no significant correlations between Machiavellianism/psychopathy subscale scores and other emotional variables.

SD3 narcissism subscale scores showed no significant correlation with emotional variables.

#### 3.6.2. For Males (Table 5)

No significant correlation was identified between SD3 and emotional variables.

## 4. Discussion

### 4.1. Associations

This study’s primary objective was to demonstrate a significant association between the scores on the validated French version of the SD3 and those on the depression, anhedonia, and alexithymia scales.

#### 4.1.1. Association Between Dark Triad and Depression

Analyses of the overall sample (males and females) showed that individuals with high scores on the SD3 also had higher depression scores on the BDI-II compared with individuals with low SD3 scores. This result aligns with the conclusions of a meta-analysis of 31 studies, which found that two of the three Dark Triad traits (psychopathy and Machiavellianism) are associated with more depressive symptoms, whereas narcissism does not show a significant association with depression (Lyu et al., 2025). This suggests that individuals with strong manipulative and antisocial tendencies report, on average, more depressive symptoms than others. This idea is supported by the findings of Al Aïn et al. (2013), who reported a positive correlation between Machiavellianism scores (MACH-IV) and total BDI-II scores (Al Aïn et al., 2013).

Another study examining all three traits simultaneously also found positive associations between psychopathy and Machiavellianism with depression, while no association was detected for narcissism (Jonason & Kroll, 2015). This latter observation is consistent with this study’s results, in which the narcissistic subscale of the SD3 showed no significant correlation with depression, anhedonia, or alexithymia. However, the SD3’s narcissism subscale mainly targets “grandiose” narcissism, characterized by a sense of superiority and self-enhancement, typically associated with high self-esteem and subjective well-being. Some studies have nuanced the concept of narcissism, demonstrating that it is not a homogeneous construct. For example, Gómez-Leal et al.’s study (2019) showed that certain “maladaptive” facets of narcissism (such as entitlement) are weakly and positively correlated with depression, whereas more adaptive facets (such as self-sufficiency) appear to correlate negatively with depression (Gómez-Leal et al., 2019). Miller et al. (2012) further distinguish between grandiose and vulnerable narcissism (Miller et al., 2012). As a brief measure, the SD3’s narcissism subscale is likely not robust enough to support future in-depth studies exploring the subtle links between narcissism subtypes and depression, anhedonia, or alexithymia.

#### 4.1.2. Association Between Dark Triad and Emotional Deficits

Emotional-processing differences (anhedonia and alexithymia) in individuals with high SD3 scores were examined compared with those with low scores. In the overall sample, individuals with high SD3 scores exhibited higher levels of alexithymia, particularly in the “difficulty identifying feelings” dimension of the TAS-20 (TAS-20 DIF). They also reported higher anhedonia scores on the BDI-II anhedonia subscale. These differences remained significant after controlling for depression, as measured with the BDI-II. These findings suggest a distinct emotional profile in individuals with Dark Triad personality traits: they struggle to clearly identify their emotions and derive little pleasure from typically rewarding activities. These results are consistent with previous studies reporting associations between the Dark Triad and related emotional deficits.

##### Regarding Alexithymia

Jonason and Krause (2013) found that each component of the Dark Triad is associated with a specific alexithymia profile. Psychopathy was correlated with difficulty describing emotions and externally oriented thinking (EOT); narcissism was associated with difficulty identifying feelings (DIF) and a lack of affective empathy; and Machiavellianism was characterized by a strong tendency toward externally oriented thinking (Gómez-Leal et al., 2019). In line with these findings, Cairncross et al. (2013) reported that alexithymia is positively correlated with psychopathic and Machiavellian traits and negatively correlated with narcissism (Cairncross et al., 2013). It, thus, appears that the more an individual exhibits manipulative and antisocial tendencies, the more likely they are to experience emotional blunting or confusion in their inner emotional life. Conversely, a grandiose narcissistic individual tends to trust their own emotional experiences and does not perceive themselves as “lacking words to describe their emotions.” High levels of Machiavellianism and psychopathy appear to be associated with elevated alexithymia, whereas narcissism shows no significant link and may even be associated with lower alexithymia, according to Cairncross et al. (2013).

##### Regarding Anhedonia

Individuals with high SD3 scores show higher levels of state anhedonia (measured with the BDI-II anhedonia subscale) than those with low SD3 scores, even after controlling for depression. This result is relatively novel, as little literature exists on the link between anhedonia and the Dark Triad. Some converging references can be found in the discussion by Gómez-Leal et al. (2019), who cite Treadway and Zald (2011) in noting that Machiavellianism is strongly associated with emotional detachment, a feature also found in depression, and that it is related to anhedonia, another common symptom of depression (Treadway & Zald, 2011). The link between Machiavellianism and anhedonia may be explained by the tendency of Machiavellian individuals toward emotional blunting and the suppression of emotional needs in favor of instrumental goals. Thus, even in the absence of clinical depression, Machiavellian individuals may experience a form of emotional coldness akin to anhedonia. These findings suggest that individuals scoring high in Dark Triad traits may lack both internal emotional insight (alexithymia; TAS-DIF) and positive affective valence (anhedonia) in their emotional experiences, even when accounting for the overall effects of depression.

### 4.2. Sex Differences

This study’s second objective was to investigate potential differences between males and females regarding the Dark Triad and its implications in terms of depression and emotional processing, as measured via alexithymia and anhedonia.

#### 4.2.1. SD3 Scores in Males and Females

Males had significantly higher SD3 scores than females across all three subdimensions of the scale. These results are consistent with previous findings (Denovan et al., 2024; Gómez-Leal et al., 2019).

#### 4.2.2. The Associations Between the Dark Triad and Emotional Deficits

A notable finding of this study was that the associations between the Dark Triad and emotional variables (measured via alexithymia and anhedonia) differed markedly by sex. When the sample was split by sex, it was found that females with high SD3 scores had significantly higher levels of depression, alexithymia (particularly on the TAS-DIF subscale), and anhedonia compared with females with low SD3 scores. These associations remained strong even after controlling for depression. Among males, however, no significant differences were observed between high and low SD3 groups in terms of emotional symptoms.

Correlation analyses confirmed this pattern: in females, the total SD3 score was positively correlated with alexithymia (the total TAS score, and especially difficulty identifying feelings) and with anhedonia, whereas no such correlations appeared in the male subgroup.

Similarly, when each Dark Triad component was examined separately, in females, both Machiavellianism and psychopathy were significantly correlated with all dimensions of alexithymia (DIF, DDF, EOT and, therefore, the total TAS score) and with anhedonia, while narcissism was not significantly correlated with any of these measures. In males, none of the three traits showed significant correlations with alexithymia, anhedonia, or depression. These findings suggest that the association between Dark Triad traits and emotional difficulties is particularly relevant among females in our sample.

These results point to a sex-based moderation effect. However, they should be interpreted with caution and considered in light of existing studies. Few have specifically examined how sex may condition the link between the Dark Triad, depression, alexithymia, and anhedonia. Gómez-Leal et al. (2019) noted that some correlations between Dark Triad subdimensions and depression varied by sex in their mixed-sex sample; for example, the narcissistic “superiority-seeking” facet was negatively correlated with depression only in females, whereas a Machiavellian “lack of morality” facet was positively correlated with depression only in males (Gómez-Leal et al., 2019). This suggests that internal dynamics associated with the same Dark Triad trait may differ between males and females.

#### 4.2.3. Hypotheses Regarding Observed Sex Differences

In this study, the complete absence of any association in males —despite their higher SD3 scores—is striking. Several biological and sociocultural explanations have been proposed.

##### Biological Factors

Testosterone, a hormone typically present at higher levels in males than in females, is associated with traits such as dominance, impulsivity, risk-taking, and a reduction in cognitive empathy and emotional reactivity. A recent study showed that the psychopathic and Machiavellian subdimensions of the Dark Triad (but not narcissism) are linked to markers of prenatal exposure to high testosterone levels. Estradiol and progesterone are also involved in emotional regulation. Specifically, estradiol is associated with improved regulation of positive emotions and offers relative protection against depression. Progesterone, particularly when elevated in the presence of low estradiol, may be linked to increased anxiety and heightened negative emotional reactivity (Albert et al., 2021).

##### Sociocultural Factors

On the one hand, males exhibit more antisocial or aggressive behavior on average, which could underlie higher psychopathy scores. This tendency toward transgression and impulsivity may have both social (e.g., greater tolerance for male aggression, and masculinity norms favoring dominance) and genetic underpinnings. On the other hand, Machiavellian traits appear to be linked to gendered social roles: adherence to traditional masculine norms (e.g., valuing competition and power) encourages manipulative and emotionally detached attitudes, which could explain male’s higher Machiavellianism scores. Krampen et al. (1990) suggested that male sex roles more strongly promote strategic deception and emotional detachment, which are core features of Machiavellianism (Krampen et al., 1990). Although we also observed higher narcissism scores among males in this study, some recent studies have not found significant overall sex differences for this trait. This may be due to a general increase in narcissism within modern populations, thereby narrowing the sex gap. However, when examining specific facets, males tend to show more vanity and entitlement, which aligns with our findings regarding total SD3 scores (Gómez-Leal et al., 2019).

One possible explanation is that males with Dark Triad traits express emotional difficulties differently than through the types of emotional processing assessed here. For instance, a man scoring high in psychopathy may externalize his distress through irritability, aggression, or risk-taking behavior rather than through complaints of sadness or anhedonia. He may, thus, experience diminished pleasure or emotional subtlety without recognizing or reporting it in a questionnaire (a sex bias in emotional expression).

In contrast, females are typically socialized to connect with their internal states and verbalize their distress more readily. As a result, a woman exhibiting manipulative or psychopathic tendencies—traits contrary to traditional sex norms—may experience greater internal conflict or social isolation, which could manifest as depressive symptoms and measurable alexithymia (Gómez-Leal et al., 2019).

##### Emotion Regulation Theory

Another relevant perspective draws on emotion regulation theory and divergent developmental trajectories. Some studies suggest that females with high psychopathic traits often have a history of emotional disturbances or early trauma, and more frequently exhibit borderline personality traits than their male counterparts. This may reflect an underlying emotional instability, which could manifest as difficulty identifying emotions (alexithymia) and chronic depressive symptoms. Males with psychopathic traits, in contrast, more often present with a “primary” cold and hardened profile characterized by fearlessness and emotional detachment, which may protect them from anxiety or overt depression. Their emotional dysfunction may also manifest in ways other than alexithymia or anhedonia, which were the focus of this study (Fournier & Verona, 2022). Garofalo et al. (2021) highlighted that emotional dysregulation—defined as the inability to manage intense affect—often mediates the relationship between psychopathic traits and aggressive behavior (Garofalo et al., 2021; Velotti et al., 2016). It could be extrapolated that, in females, this dysregulation may also lead to internalizing symptoms (depression, anhedonia, and alexithymia) in addition to behavioral externalization.

In this study, the lack of association in males between SD3 scores and depression, alexithymia, or anhedonia does not imply that males with Dark Triad traits lack vulnerability. Rather, these vulnerabilities may manifest in forms other than anhedonia, alexithymia, or depression, such as substance abuse or risky behaviors.

### 4.3. Limitations

Several limitations must be considered when interpreting this study’s results. First, this was a cross-sectional design, which does not allow for causal conclusions to be drawn between Dark Triad traits, emotional symptoms, and sociodemographic variables. The observed associations should, therefore, be interpreted as correlational rather than indicative of any directional effect.

Moreover, all data were collected via self-administered online questionnaires, with no clinical or behavioral assessment. This introduces potential biases, including social desirability, variable understanding of certain items, or insincere responses, although anonymity likely reduced the latter risk. Participants were not formally assessed for psychiatric diagnoses. While the scales used are validated and widely employed, they cannot substitute for structured clinical evaluations. Furthermore, although this study’s results were adjusted for the severity of depressive symptoms, given their central role in the occurrence of alexithymia and anhedonia for the general population (Honkalampi et al., 2000; Damerdji et al., 2022), the failure to account for other pre-existing or underlying behavioral and/or psychological factors may limit the interpretation and generalization of our results.

Recruitment was conducted online, primarily via the social media platform Facebook in 2016, which may have introduced a selection bias: participants who chose to respond are likely to be more comfortable with digital tools, more introspective, and possibly not representative of the general population. The general population and Facebook features cannot be expected to be distributed similarly. Similarly, as part of the sample was recruited via university mailing lists, the resulting population may overrepresent individuals with higher educational levels, limiting the generalizability of the findings to the broader general population. The male sample (*N* = 169) was smaller than the female sample (*N* = 323), which may limit the generalizability of the findings to the male population. This imbalance may have affected our ability to detect associations in the male subsample, despite sample sizes respecting the conditions of use of the statistical tests used, indicating that the lack of significant findings among males might reflect insufficient statistical power rather than true sex differences. However, the use of non-parametric tests in this study may have limited as much as possible this risk of potential bias in our results induced by this difference in sample size between males and females, since in case of the impossibility of using parametric tests, the non-parametric tests are very robust to detect differences, even in the case of smaller sample sizes (Fay & Proschan, 2010; Okeh, 2009). Finally, recent research also suggests that females tend to participate more readily and promptly in online surveys than males, potentially contributing to lower male representation in our sample (Becker, 2022).

While the instruments used are validated, some of them (e.g., the SD3 and TAS-20) are relatively broad and do not allow for a fine-grained exploration of the clinical and developmental dimensions associated with Dark Triad personality traits.

Furthermore, although the original version of the SD3 has demonstrated high test–retest reliability, this property has not yet been evaluated for the French translation used in this study. In addition, since the SD3 primarily targets “grandiose” narcissism, this scale does not allow for the assessment of other dimensions of narcissism (such as “vulnerable” narcissism). These limitations underscore the need for further complementary studies—ideally longitudinal and clinically anchored—that could explore the temporal dynamics of the observed associations and their validity in more structured diagnostic and/or therapeutic contexts.

Finally, an important limitation of this study concerns the binary treatment of sex (males/females). Our study sought to explore sex differences between males and females as a first step, before addressing the specific situation of non-binary populations, which warrants dedicated methodological and conceptual frameworks. While sex emerged as a key variable in our analyses, our dataset—created in 2015–2016—did not include the option for participants to identify outside of a binary framework. This reflects a limitation in research practices at the time of data collection and not an assumption about the sex binary as an exhaustive model. We recognize that gender identity exists on a spectrum, and future studies on the Dark Triad and emotional functioning should adopt more inclusive designs to ensure adequate representation and analysis of non-binary and gender-diverse individuals. The psychological experiences and emotional vulnerabilities of non-binary populations remain insufficiently studied in this field and deserve careful attention.

## 5. Conclusions

This study’s results offer new insights into the associations between Dark Triad traits, depression, alexithymia, and anhedonia in an adult French-speaking sample. They reinforce the validity of the French validated SD3 scale. In line with the existing literature, the findings confirm that Machiavellianism and psychopathy—but not grandiose narcissism—are associated with depressive symptoms and specific emotional deficits, particularly difficulty identifying emotions (alexithymia; TAS-DIF) and a reduced capacity to experience pleasure (anhedonia), even after adjusting for depression.

The present sample showed that these associations are not uniform across sex: they are clearly present in females but absent in males. These differences may be explained by biological (hormonal and neuroendocrine) factors, sociocultural influences related to sex roles, and differing modes of emotional regulation.

Although Dark Triad traits may appear socially “adaptive” for some individuals, they are accompanied in others—particularly females—by notable emotional vulnerability. These findings underscore the need for a nuanced, sex-sensitive approach in both research and clinical understanding of individuals with Dark Triad personality traits. In females, traits such as Machiavellianism and psychopathy may be accompanied by greater emotional distress, in the form of alexithymia and anhedonia, regardless of overall depressive severity. Furthermore, the use of the SD3 primarily targeting “grandiose” narcissism may have masked a potential impact of narcissism on the occurrence of emotional distress in females, since, unlike males, females more frequently present “vulnerable” narcissism associated with greater emotional fragility. This supports an integrative therapeutic approach that addresses mood symptoms alongside emotional awareness and regulation, which are often impaired in such profiles. Techniques drawn from schema therapy, mentalization-based therapy, or mindfulness-based interventions may be relevant to enhance emotional awareness and restore the capacity for pleasure.

Conversely, in males with high levels of dark traits, the absence of reported emotional distress should not obscure possible externalized problems (e.g., substance use, impulsivity, and social withdrawal), which require careful assessment. These findings call for a sex-informed individualization of clinical interviews and therapeutic alliances to better identify the underlying needs beneath seemingly “cold” or defensive personality profiles.

Future research—especially longitudinal and qualitative studies—will be essential to further explore the underlying mechanisms of these associations and their clinical implications.

## Data Availability

The datasets used and/or analyzed during the current study are available from the corresponding author on reasonable request.

## Supplementary Materials

The following supporting information can be downloaded at https://www.mdpi.com/article/10.3390/bs15101369/s1, Supplementary Data S1: Psychometric properties of the French version of the Short Dark Triad (SD3); Supplementary Data S2: Detailed description of the different variables studied according to age ranges; Supplementary Data S3: Adjusted comparative analyses for whole sample, females and males.

## References

[B1-behavsci-15-01369] Adewuya A. O., Coker O. A., Atilola O., Ola B. A., Zachariah M. P., Adewumi T., Olugbile O., Fasawe A., Idris O. (2018). Gender difference in the point prevalence, symptoms, comorbidity, and correlates of depression: Findings from the Lagos State Mental Health Survey (LSMHS), Nigeria. Archives of Women’s Mental Health.

[B2-behavsci-15-01369] Al Aïn S., Carré A., Fantini-Hauwel C., Baudouin J. Y., Besche-Richard C. (2013). What is the emotional core of the multidimensional Machiavellian personality trait?. Frontiers in Psychology.

[B3-behavsci-15-01369] Albert K. M., Boyd B. D., Taylor W. D., Newhouse P. A. (2021). Differential effects of estradiol on neural and emotional stress response in postmenopausal women with remitted major depressive disorder. Journal of Affective Disorders.

[B4-behavsci-15-01369] Amerio A., Natale A., Gnecco G. B., Lechiara A., Verrina E., Bianchi D., Fusar-Poli L., Costanza A., Serafini G., Amore M., Aguglia A. (2023). The role of gender in patients with borderline personality disorder: Differences related to hopelessness, alexithymia, coping strategies, and sensory profile. Medicina.

[B5-behavsci-15-01369] Beck A. T., Steer R. A., Ball R., Ranieri W. (1996). Comparison of beck depression inventories–IA and –II in psychiatric outpatients. Journal of Personality Assessment.

[B6-behavsci-15-01369] Becker R. (2022). Gender and survey participation: An event history analysis of the gender effects of survey participation in a probability-based multi-wave panel study with a sequential mixed-mode design. Methods, Data, Analyses.

[B7-behavsci-15-01369] Book A., Visser B. A., Volk A. A. (2015). Unpacking “evil”: Claiming the core of the Dark Triad. Personality and Individual Differences.

[B8-behavsci-15-01369] Brugués G., Caparrós B. (2021). Dysfunctional personality, dark triad and moral disengagement in incarcerated offenders: Implications for recidivism and violence. Psychiatry, Psychology and Law.

[B9-behavsci-15-01369] Cairncross M., Veselka L., Schermer J. A., Vernon P. A. (2013). A behavioral genetic analysis of alexithymia and the dark triad traits of personality. Twin Research and Human Genetics.

[B10-behavsci-15-01369] Coleman S. R. M., Pincus A. L., Smyth J. M. (2019). Narcissism and stress-reactivity: A biobehavioural health perspective. Health Psychology Review.

[B11-behavsci-15-01369] Conenna M., Point C., Wacquier B., Lanquart J. P., Hein M. (2025). Risk of major depression associated with excessive daytime sleepiness in apneic individuals. Clocks & Sleep.

[B12-behavsci-15-01369] Damerdji F., Rotsaert M., Wacquier B., Hein M., Loas G. (2022). Prevalence and relationships between alexithymia, anhedonia, depression and anxiety during the Belgian COVID-19 pandemic lockdown. International Journal of Environmental Research and Public Health.

[B13-behavsci-15-01369] Denovan A., Plouffe R. A., Dagnall N., Artamonova E., Kowalski M. C., Saklofske D. H. (2024). Dark triad, dyad, or core? A psychometric evaluation of the Short Dark Triad (SD3) across three countries. Current Psychology.

[B14-behavsci-15-01369] Dragostinov Y., Mõttus R. (2023). Test-Retest reliability and construct validity of the brief dark triad measurements. Journal of Personality Assessment.

[B15-behavsci-15-01369] Duarte A. C., Matos A. P., Marques C. (2015). Cognitive emotion regulation strategies and depressive symptoms: Gender’s moderating effect. Procedia-Social and Behavioral Sciences.

[B16-behavsci-15-01369] Fay M. P., Proschan M. A. (2010). Wilcoxon-Mann-Whitney or t-test? On assumptions for hypothesis tests and multiple interpretations of decision rules. Statistics Surveys.

[B17-behavsci-15-01369] Fournier L. F., Verona E., Vitale J. E. (2022). Psychopathy, trauma, and PTSD symptoms: Theory and evidence. The complexity of psychopathy.

[B18-behavsci-15-01369] French D. C., Lanquart J.-P., Rotsaert M., Loas G. (2024). Validation of the French version of the Short Dark Triad (SD3). Annales Medico-Psychologiques.

[B19-behavsci-15-01369] Garofalo C., Neumann C. S., Velotti P. (2021). Psychopathy and aggression: The role of emotion dysregulation. Journal of Interpersonal Violence.

[B20-behavsci-15-01369] Garofalo C., Virgilio C., Bogaerts S., Schimmenti A. (2019). Dark ladies: Maladaptive personality domains, alexithymia, and the dark triad in women. Clinical Neuropsychiatry.

[B21-behavsci-15-01369] Gómez-Leal R., Gutiérrez-Cobo M. J., Megías-Robles A., Fernández-Berrocal P. (2023). The dark triad and subjective well-being: The mediating role of cognitive-emotional regulation strategies. Scandinavian Journal of Psychology.

[B22-behavsci-15-01369] Gómez-Leal R., Megías-Robles A., Gutiérrez-Cobo M. J., Cabello R., Fernández-Abascal E. G., Fernández-Berrocal P. (2019). Relationship between the dark triad and depressive symptoms. PeerJ.

[B23-behavsci-15-01369] Green A., MacLean R., Charles K. (2022). Female narcissism: Assessment, aetiology, and behavioural manifestations. Psychological Reports.

[B24-behavsci-15-01369] Grigoras M., Wille B. (2017). Shedding light on the dark side: Associations between the dark triad and the DSM-5 maladaptive trait model. Personality and Individual Differences.

[B25-behavsci-15-01369] Grijalva E., Newman D. A., Tay L., Donnellan M. B., Harms P. D., Robins R. W., Yan T. (2015). Gender differences in narcissism: A meta-analytic review. Psychological Bulletin.

[B26-behavsci-15-01369] Hampejs V., Zwickl A. A., Tran U. S., Voracek M. (2025). The dark triad of personality and criminal and delinquent behavior: Preregistered systematic review and three-level meta-analysis. Personality and Individual Differences.

[B27-behavsci-15-01369] Honkalampi K., Hintikka J., Tanskanen A., Lehtonen J., Viinamäki H. (2000). Depression is strongly associated with alexithymia in the general population. Journal of Psychosomatic Research.

[B28-behavsci-15-01369] Hurezan L., Turi A., Ion A., Visu-Petra L. (2024). Dark and bright personality dimensions as predictors of criminal behavior and recidivism. Scientific Reports.

[B29-behavsci-15-01369] Joiner T. E., Brown J. S., Metalsky G. I. (2003). A test of the tripartite model’s prediction of anhedonia’s specificity to depression: Patients with major depression versus patients with schizophrenia. Psychiatry Research.

[B30-behavsci-15-01369] Jonason P. K., Baughman H. M., Carter G. L., Parker P. (2020). Country-level correlates of the Dark Triad traits in 49 countries. Journal of Personality.

[B31-behavsci-15-01369] Jonason P. K., Krause L. (2013). The emotional deficits associated with the dark triad traits: Cognitive empathy, affective empathy, and alexithymia. Personality and Individual Differences.

[B32-behavsci-15-01369] Jonason P. K., Kroll C. H. (2015). A multidimensional view of the relationship between empathy and the dark triad. Journal of Individual Differences.

[B33-behavsci-15-01369] Jones D. N., Paulhus D. L. (2014). Introducing the short dark triad (SD3): A brief measure of dark personality traits. Assessment.

[B34-behavsci-15-01369] Kowalski C. M., Rogoza R., Saklofske D. H., Schermer J. A. (2021). Dark triads, tetrads, tents, and cores: Why navigate (research) the jungle of dark personality models without a compass (criterion)?. Acta Psychologica.

[B35-behavsci-15-01369] Krampen G., Effertz B., Jostock U., Müller B. (1990). Gender differences in personality: Biological and/or psychological?. European Journal of Personality.

[B36-behavsci-15-01369] Li J., Liu C., Albertella L., Rotaru K., Li K., Zhou Y., Wei X., Yuan S., Liu X., Ren L. (2024). Network analysis of the association between dark triad traits and depression symptoms in university students. Personality and Individual Differences.

[B37-behavsci-15-01369] Li S., Zhang B., Guo Y., Zhang J. (2015). The association between alexithymia as assessed by the 20-item Toronto Alexithymia Scale and depression: A meta-analysis. Psychiatry Research.

[B38-behavsci-15-01369] Loas G., Monestes J. L., Ameller A., Bubrovszky M., Yon V., Wallier J., Berthoz S., Corcos M., Thomas P., Gard D. E. (2009). Psychometric properties of the French version of the Temporal Experience of Pleasure Scale (TEPS): Study on 125 university students and on 162 psychiatric subjects. Annales Medico-Psychologiques.

[B39-behavsci-15-01369] Loas G., Parker J. D., Otmani O., Verrier A., Fremaux D. (1997). Confirmatory factor analysis of the French translation of the 20-item Toronto Alexithymia Scale. Perceptual and Motor Skills.

[B40-behavsci-15-01369] Loeffler L. A. K., Huebben A. K., Radke S., Habel U., Derntl B. (2020). The association between vulnerable/grandiose narcissism and emotion regulation. Frontiers in Psychology.

[B41-behavsci-15-01369] Luo Y. L. L., Kovas Y., Wang L., Stalikas A., Kyriazos T. A., Gianniou F.-M., Likhanov M. V., Papageorgiou K. A. (2023). Sex differences in the dark triad are sensitive to socioeconomic conditions: The adaptive value of narcissism in the UK, Greece, and China. Current Psychology.

[B42-behavsci-15-01369] Luque B., Castillo-Mayén R., Cuadrado E., Gutiérrez-Domingo T., Rubio S. J., Arenas A., Delgado-Lista J., Pérez Martínez P., Tabernero C. (2020). The role of emotional regulation and affective balance on health perception in cardiovascular disease patients according to sex differences. Journal of Clinical Medicine.

[B43-behavsci-15-01369] Lyu C., Xu D., Chen G. (2025). Dark and blue: A meta-analysis of the relationship between dark triad and depressive symptoms. Journal of Research in Personality.

[B44-behavsci-15-01369] Maples J. L., Lamkin J., Miller J. D. (2014). A test of two brief measures of the dark triad: The dirty dozen and short dark triad. Psychological Assessment.

[B45-behavsci-15-01369] Mendia J., Zumeta L. N., Cusi O., Pascual A., Alonso-Arbiol I., Díaz V., Páez D. (2024). Gender differences in alexithymia: Insights from an updated meta-analysis. Personality and Individual Differences.

[B46-behavsci-15-01369] Miller J. D., Price J., Gentile B., Lynam D. R., Campbell W. K. (2012). Grandiose and vulnerable narcissism from the perspective of the interpersonal circumplex. Personality and Individual Differences.

[B47-behavsci-15-01369] Mojsa-Kaja J., Szklarczyk K., González-Yubero S., Palomera R. (2021). Cognitive emotion regulation strategies mediate the relationships between dark triad traits and negative emotional states experienced during the COVID-19 pandemic. Personality and Individual Differences.

[B48-behavsci-15-01369] Muris P., Merckelbach H., Otgaar H., Meijer E. (2017). The malevolent side of human nature: A meta-analysis and critical review of the literature on the dark triad (narcissism, Machiavellianism, and psychopathy). Perspectives on Psychological Science.

[B49-behavsci-15-01369] Navas M. P., Maneiro L., Cutrín O., Gómez-Fraguela J. A., Sobral J. (2021). Contributions of the dark triad to moral disengagement among incarcerated and community adults. Legal and Criminological Psychology.

[B50-behavsci-15-01369] Nolen-Hoeksema S. (2012). Emotion regulation and psychopathology: The role of gender. Annual Review of Clinical Psychology.

[B51-behavsci-15-01369] Okeh U. M. (2009). Statistical analysis of the application of Wilcoxon and Mann-Whitney U test in medical research studies. Biotechnology and Molecular Biology Reviews.

[B52-behavsci-15-01369] Papageorgiou K. A., Denovan A., Dagnall N., Hill-Artamonova E., Gianniou F. M., Papageorgiou S., Plouffe R. A., Kowalski C. M., Saklofske D. H., Kyriazos T., Stalikas A., Costantini G. (2023). Grandiose narcissism indirectly associates with lower psychopathology across five countries. Journal of Psychiatric Research.

[B53-behavsci-15-01369] Parker G., Fletcher K., Paterson A., Anderson J., Hong M. (2014). Gender differences in depression severity and symptoms across depressive sub-types. Journal of Affective Disorders.

[B54-behavsci-15-01369] Paulhus D. L., Williams K. M. (2002). The Dark Triad of personality: Narcissism, Machiavellianism and psychopathy. Journal of Research in Personality.

[B55-behavsci-15-01369] Pechorro P., Curtis S., DeLisi M., Maroco J., Nunes C. (2022). Dark triad psychopathy outperforms self-control in predicting antisocial outcomes: A structural equation modeling approach. European Journal of Investigation in Health, Psychology and Education.

[B56-behavsci-15-01369] Pu J., Liu Y., Ren X. (2025). The potential roles of social ostracism and loneliness in the development of Dark Triad traits in adolescents: A longitudinal study. Journal of Personality.

[B57-behavsci-15-01369] Ricarte Trives J. J., Navarro Bravo B., Latorre Postigo J. M., Ros Segura L., Watkins E. (2016). Age and gender differences in emotion regulation strategies: Autobiographical memory, rumination, problem solving and distraction. The Spanish Journal of Psychology.

[B58-behavsci-15-01369] Sabouri S., Gerber M., Lemola S., Becker S. P., Shamsi M., Shakouri Z., Sadeghi Bahmani D., Kalak N., Holsboer-Trachsler E., Brand S. (2016). Examining dark triad traits in relation to sleep disturbances, anxiety sensitivity and intolerance of uncertainty in young adults. Comprehensive Psychiatry.

[B59-behavsci-15-01369] Schmitt D. P., Long A. E., McPhearson A., O’Brien K., Remmert B., Shah S. H. (2017). Personality and gender differences in global perspective. International Journal of Psychology.

[B60-behavsci-15-01369] Schreiber M., Sellbom M., Marcus D. K. (2020). The place of the “Dark Triad” in general models of personality: Some meta-analytic clarification. Psychological Bulletin.

[B61-behavsci-15-01369] Serretti A. (2023). Anhedonia and depressive disorders. Clinical Psychopharmacology and Neuroscience.

[B62-behavsci-15-01369] Shen K. (2022). The dark triad and depressive symptoms among Chinese adolescents: Moderated mediation models of age and emotion regulation strategies. Current Psychology.

[B63-behavsci-15-01369] Treadway M. T., Zald D. H. (2011). Reconsidering anhedonia in depression: Lessons from translational neuroscience. Neuroscience & Biobehavioral Reviews.

[B64-behavsci-15-01369] Velotti P., Garofalo C., Petrocchi C., Cavallo F., Popolo R., Dimaggio G. (2016). Alexithymia, emotion dysregulation, impulsivity and aggression: A multiple mediation model. Psychiatry Research.

[B65-behavsci-15-01369] Welkie J., Babinski D. E., Neely K. A. (2021). Sex and emotion regulation difficulties contribute to depression in young adults with attention-deficit/hyperactivity disorder. Psychological Reports.

