# Supplementary material for: Dark Triad, Depression, Anhedonia and Alexithymia: The Role of Sex Differences
Source: Behav Sci (Basel). 2025 Oct 7;15(10):1369. doi: 10.3390/bs15101369 (PMC12561530; doi:10.3390/bs15101369)
Supplement: Supplementary file 1 [file behavsci-15-01369-s001.zip › behavsci-3806421-supplementary.pdf]

Supplementary Data S1 – Psychometric properties of the French version of the Short Dark Triad (SD3)

Table S1: Cronbach's alpha for Short Dark Triad (SD3) (n=492)

	Cronbach's alpha
SD3 – Machiavellianism	0.79
SD3 – Narcissism	0.69
SD3 – Psychopathy	0.68
SD3 – Total	0.85

Table S2: Confirmatory factor analysis for Short Dark Triad (SD3) (3 factors: Machiavellianism, narcissism and psychopathy) (n=492)

	3-factor correlated model	Interpretation
GFI (≥0.85)	0.91	Good fit
RMSEA (<0.08)	0.06	Good fit
CFI (>0.90)	0.80	Acceptable fit
SRMR (<0.08)	0.06	Good fit

GFI = Goodness of Fit Index, RMSEA = Root Mean Square Error of Approximation, CFI = Comparative Fit Index, SRMR = Standardized Root Mean Square Residual

Supplementary Data S2 – Detailed description of the different variables studied according to age ranges

Table S1: Detailed description of the different variables studied according to age ranges for whole sample (n=492)

Variables	Young adult (n=85) (18-25 years)	Adult (n=342) (25-40 years)	Middle-aged to older adult (n=65) (>40 years)	P-value
SD3 Variables				
SD3 – Machiavellianism	29 (25 – 33)	28 (24 – 33)	27 (23 – 30)	0.039[b]
SD3 – Narcissism	24 (21 – 27)	24 (22 – 28)	25 (22 – 29)	0.656
SD3 – Psychopathy	20 (17 – 23)	20 (17 – 23)	19 (16 – 23)	0.480
SD3 – Total	75 (65 – 84)	72 (65 – 81)	72 (62 – 81)	0.488
Emotional variables				
TAS – DIF	19 (14 – 24)	17 (13 -22)	15 (13 – 18)	0.002[b]
TAS – DDF	15 (12 – 21)	13 (10 – 18)	13 (10 – 15)	<0.001[a,b]
TAS – EOT	17 (15 – 22)	16 (13 – 19)	16 (14 – 19)	0.260
TAS – Total	53 (44 – 59)	48 (39 – 56)	43 (38 – 51)	<0.001[a,b]
Trait anhedonia – Anticipatory	5 (5 – 6)	5 (5 – 6)	5 (5 – 6)	0.963
Trait anhedonia – Consummatory	6 (5 – 8)	6 (5 – 8)	6 (5 – 7)	0.115
Trait anhedonia – Total	11 (10 – 13)	12 (10 – 13)	11 (10 – 13)	0.283
Recent change of anhedonia	1 (0 – 2)	1 (0 – 2)	1 (0 – 2)	0.969
Confounding variables				
Age (years)	23 (22 – 24)	29 (27 – 32)	48 (43 – 56)	<0.001[a,b,c]
Sex (M)	37.7%	32.8%	38.5%	0.526
BDI – Total	12 (6 – 20)	10 (5 – 17)	10 (4 – 15)	0.270

SD3 = Short Dark Triad, TAS = Toronto Alexithymia Scale, DIF = Difficulty identifying feelings, DDF = Difficulty describing feelings, EOT = Externally-oriented thinking, BDI = Beck Depression Inventory.

[a] Young adult vs adult, [b] Young adult vs Middle-aged to older adult, [c] Adult vs Middle-aged to older adult

Table S2: Detailed description of the different variables studied according to age ranges for females (n=323)

Variables	Young adult (n=53) (18-25 years)	Adult (n=230) (25-40 years)	Middle-aged to older adult (n=40) (>40 years)	P-value
SD3 Variables				
SD3 – Machiavellianism	26 (23 – 31)	27 (23 – 31)	26 (22 – 30)	0.183
SD3 – Narcissism	23 (18 – 26)	24 (21 – 27)	24 (21 – 28)	0.208
SD3 – Psychopathy	19 (17 – 23)	19 (15 – 22)	19 (16 – 21)	0.523
SD3 – Total	69 (61 – 79)	70 (63 – 77)	70 (59 – 78)	0.747
Emotional variables				
TAS – DIF	20 (14 – 24)	17 (13 -22)	15 (14 – 18)	0.030[b]
TAS – DDF	14 (10 – 20)	13 (10 – 18)	13 (9 – 15)	0.043[a,b]
TAS – EOT	17 (15 – 20)	16 (13 – 18)	16 (13 – 19)	0.075
TAS – Total	51 (43 – 59)	47 (38 – 55)	43 (38 – 50)	0.011[a,b]
Trait anhedonia – Anticipatory	5 (5 – 6)	5 (5 – 6)	5 (5 – 6)	0.996
Trait anhedonia – Consummatory	6 (5 – 7)	6 (5 – 7)	6 (5 – 7)	0.079
Trait anhedonia – Total	11 (10 – 13)	12 (10 – 13)	11 (10 – 13)	0.249
Recent change of anhedonia	1 (0 – 2)	1 (0 – 2)	1 (0 – 2)	0.380
Confounding variables				
Age (years)	23 (22 – 24)	29 (27 – 32)	52 (44 – 58)	<0.001[a,b,c]
BDI – Total	11 (6 – 18)	11 (5 – 18)	11 (3 – 16)	0.503

SD3 = Short Dark Triad, TAS = Toronto Alexithymia Scale, DIF = Difficulty identifying feelings, DDF = Difficulty describing feelings, EOT = Externally-oriented thinking, BDI = Beck Depression Inventory.

[a] Young adult vs adult, [b] Young adult vs Middle-aged to older adult, [c] Adult vs Middle-aged to older adult

Table S3: Detailed description of the different variables studied according to age ranges for males (n=169)

Variables	Young adult (n=32) (18-25 years)	Adult (n=112) (25-40 years)	Middle-aged to older adult (n=25) (>40 years)	P-value
SD3 Variables				
SD3 – Machiavellianism	33 (30 – 38)	31 (26 – 36)	28 (27 – 30)	0.003[a,b]
SD3 – Narcissism	26 (24 – 32)	25 (23 – 30)	27 (23 – 30)	0.716
SD3 – Psychopathy	22 (20 – 25)	22 (19 – 26)	22 (17 – 25)	0.469
SD3 – Total	81 (76 – 91)	78 (71 – 87)	75 (68 – 82)	0.097
Emotional variables				
TAS – DIF	19 (16 – 24)	17 (14 -21)	15 (13 – 18)	0.050
TAS – DDF	17 (15 – 22)	15 (11 – 19)	13 (11 – 16)	0.002[a,b]
TAS – EOT	16 (14 – 19)	17 (14 – 19)	16 (15 – 17)	0.922
TAS – Total	54 (47 – 59)	49 (40 – 58)	44 (39 – 52)	0.018[b]
Trait anhedonia – Anticipatory	5 (4 – 6)	5 (4 – 6)	5 (4 – 6)	0.962
Trait anhedonia – Consummatory	8 (5 – 9)	6 (5 – 8)	6 (5 – 7)	0.096
Trait anhedonia – Total	13 (10 – 14)	11 (10 – 13)	11 (10 – 13)	0.198
Recent change of anhedonia	2 (0 – 3)	1 (0 – 2)	1 (0 – 2)	0.160
Confounding variables				
Age (years)	23 (22 – 24)	30 (28 – 31)	46 (42 – 51)	<0.001[a,b,c]
BDI – Total	14 (6 – 22)	9 (5 – 15)	10 (7 – 15)	0.123

SD3 = Short Dark Triad, TAS = Toronto Alexithymia Scale, DIF = Difficulty identifying feelings, DDF = Difficulty describing feelings, EOT = Externally-oriented thinking, BDI = Beck Depression Inventory.
[a] Young adult vs adult, [b] Young adult vs Middle-aged to older adult, [c] Adult vs Middle-aged to older adult

Supplementary Data S3 – Adjusted comparative analyses for whole sample, females and males

Table S1: Adjusted comparative analyses for whole sample (n=492)

Variables	b_{a1}(ES)<br>Low SD3 scores vs. high SD3 scores
Emotional variables	
TAS – DIF	2.0 (0.8)[a]
TAS – DDF	1.0 (0.6)
TAS – EOT	0.0 (0.5)
TAS – Total	4.0 (1.3)[a]
Trait anhedonia – Anticipatory	0.0 (0.1)
Trait anhedonia – Consummatory	0.0 (0.2)
Trait anhedonia – Total	0.0 (0.2)
Recent change of anhedonia	1.0 (0.1)[a]
	[a] $p<0.05$

b_{a1} (ES): quantile regression coefficient adjusted (standard error). These coefficients are the difference of median between females + males with low SD3 scores and females + males with high SD3 scores, adjusted for depression severity.

SD3 = Short Dark Triad, TAS = Toronto Alexithymia Scale, DIF = Difficulty identifying feelings, DDF = Difficulty describing feelings, EOT = Externally-oriented thinking, BDI = Beck Depression Inventory.

Table S2: Adjusted comparative analyses for females (n=323)

**Variables**	**b_{a1}(ES)**<br>**Low SD3 scores vs. high SD3 scores**
Emotional variables	
TAS – DIF	3.0 (0.9)[a]
TAS – DDF	1.0 (0.7)
TAS – EOT	0.0 (0.6)
TAS – Total	4.0 (1.6)[a]
Trait anhedonia – Anticipatory	0.0 (0.2)
Trait anhedonia – Consummatory	0.0 (0.3)
Trait anhedonia – Total	0.0 (0.3)
Recent change of anhedonia	1.0 (0.2)[a]
	[a] $p<0.05$

b_{a1} (ES): quantile regression coefficient adjusted (standard error). These coefficients are the difference of median between females with low SD3 scores and females with high SD3 scores, adjusted for depression severity.

SD3 = Short Dark Triad, TAS = Toronto Alexithymia Scale, DIF = Difficulty identifying feelings, DDF = Difficulty describing feelings, EOT = Externally-oriented thinking, BDI = Beck Depression Inventory.

Table S3: Adjusted comparative analyses for males (n=169)

Variables	b_{a1}(ES)<br>Low SD3 scores vs. high SD3 scores
Emotional variables	
TAS – DIF	2.0 (1.1)
TAS – DDF	0.0 (1.1)
TAS – EOT	0.0 (0.7)
TAS – Total	2.0 (2.2)
Trait anhedonia – Anticipatory	0.0 (0.2)
Trait anhedonia – Consummatory	0.0 (0.3)
Trait anhedonia – Total	1.0 (0.6)
Recent change of anhedonia	1.0 (0.7)
	[a] $p<0.05$

b_{a1} (ES): quantile regression coefficient adjusted (standard error). These coefficients are the difference of median between males with low SD3 scores and males with high SD3 scores, adjusted for depression severity.

SD3 = Short Dark Triad, TAS = Toronto Alexithymia Scale, DIF = Difficulty identifying feelings, DDF = Difficulty describing feelings, EOT = Externally-oriented thinking, BDI = Beck Depression Inventory.
